# Mr. Cuming, on Leprosy

**Published:** 1804-11-01

**Authors:** Ralph Cuming

**Affiliations:** Romsey, Hants


### *To the Editors of the Medical and Physical Journal.*

GENTLEMEN,

FROM a supposition that the successful treatment of a Case of Leprosy may not be unacceptable to the readers of the Medical Journal, I beg leave to offer to their notice a brief account of the disease, with the remedies employed, and the reflections resulting therefrom.

A boy, the son of a *Hampshire* farmer, 15 years of age, had for the space of six months previously to his application to me, been subject to violent itching in different parts of his body; and when I saw him, his body and limbs were thickly covered with scales of a scabby scurfy nature, which when removed, the parts underneath exhibited an inflamed surface, of a hue inclining to copper colour. Supposing from the history of the case, as related by his parents, that his disease had been either occasioned, or greatly aggravated, by his mode of living, which it appeared had consisted chiefly of smoke-dried and salted provisions, change of diet was naturally the first indication of cure that presented itself; I therefore desired he might be allowed a sufficient quantity of fresh animal food, fruits, and vegetables, and prescribed a calomel purge, with the following alterative boluses and ointment, ℞. Calomel ppt. gr. ij. Pulv. antimonialis, gr. ix. Opii. gr. iß. Cons. rosa. q. s. ft. Bol. no. ix. Capt. j. ter de die. ℞. Hydrarg. muriat. gr. viij. Adipis suilla ℥ij. M. ft. Ungt. part afft. applicandum. nocte maneque. By persevering in the use of these remedies, with little variation, for the space of six weeks, the white scurfy scales intirely disappeared, and the subjacent parts began to assume their natural colour; the only thing that now remained to be effected was the cure of several veral deep fissures in the palms of his hands, which I considered as being truly characteristic of the disease, and frequently a concomitant symptom; but the remedies so eminently useful in curing the other morbid parts, had no power over these fissures; and after trying argent. nitrat. with a variety of other means, I was obliged to have recourse to blistering, which had the wished-for effect, producing such a degree of excitement on the cutis vera, as to enable it to throw off the diseased action that it had been so long accustomed to, and which rendered it necessary to employ stimulating dressings.

This is the only case of leprosy which I have had under my care, except one that occurred during my public services; and the hands were, in this case also, the most difficult to manage, which I think would never have been cured without blistering. From the history of the boy's case, it would appear that unwholesome food was in some measure the remote cause of his complaint; and though the cutis vera is the seat of this disease, and may therefore be considered as partaking more of the nature of a local than general affection, yet such medicines as have a tendency to promote the secretions, and produce alterative effects on the mass of humours, must consequently be acknowledged as useful auxiliaries. The scabrous incrustations, so shocking to the eye of the spectator, and hateful to the patient, I conceive to be occasioned by rubbing and scratching the cuticular excretory vessels, which being wounded, pour out their contents on the surface. If this be the case, it will appear evident, that stimulating applications, or such as allay the itching sensations, and produce those which are diametrically opposite, or painful, must have a good effect, by immediately striking at the root of the evil, and relieving the patient from the disagreeable necessity of using his nails; but in all inveterate cases, when the skin has acquired an unusual degree of thickness, the warm bath will certainly become absolutely requisite; and the Bath waters, so famous for cutaneous diseases, may have a two-fold virtue, by cleansing and softening the skin, as well as stimulating it, thereby enabling the absorbents to carry off that surcharge or redundancy of fluids, which have been collected on the surface. An objection may be made to this mode of procedure, on a supposition that turning the current of humours, which Nature seems to have directed to the skin, may eventually produce complaints more terrific in their consequences; but for my part, I should conceive such an opinion to be merely chimerical; and I mention it because it appears to me, the only one that can be urged against a practice, which I consider both safe and salutary, when the habit is every day undergoing some change by means of proper regimen. And however specious such reasoning may appear, it can never be advanced as an established theorem, whilst the practice recommended has the broad basis of experience for its foundation.

During my studies at a celebrated medical school, the flour bag was the only application then in vogue for Erysipelas, from an idea that it was dangerous to employ refrigerants; medicines of this class are frequently termed repellents, and it would have been a happy circumstance for many patients, if their medical attendants had not been frightened out of their wits, at the thought of this terrible name *repellent!* The famous Cullen, when treating on Erysipelas, seems to conceive that no other application, save meal or flour, is admissible, except when there is a sense of throbbing in the inflamed part, indicating suppuration; yet I must dissent from such an opinion, though sanctioned by so great a writer, in this instance; for if the refrigerant plan was resorted to in the early stages of the disease, suppuration would rarely happen. The Doctor does not speak in positive terms with respect to the nature of this disease, that is to say, whether it be local or constitutional; yet he says, he never knew a translation of the inflammation from the limbs to other parts; and when it happens that the affection of the face is communicated to the brain, he supposes it to be entirely caused by the spreading of the inflammation. Does not this, at once point out the necessity and propriety of having recourse to refrigerants, without leaving the work entirely to Nature, and trusting to flour as the only local application? My practice, in every case of erysipelas, has been invariably the following, which I shall endeavour to illustrate by annexing a case that occurred to me the other day. I was desired to visit the wife of a labouring man, whom I found with a shocking erysipelatous inflammation in her face; her pulse, full and frequent; in fine, she was in a complete state of pyrexia. I immediately bled her, sent a cathartic with a refrigerant lotion, composed of a weak solution of cerussa acetata, desiring that her face might be kept constantly covered with wet linen rags, and renewed as often, and as cold as possible. By this treatment, her face, in less than 24 hours, was free from inflammation; and her eyes, which before were perfectly closed with the swelling, were now restored to their natural state. Hence it appears how extremely fallacious theories are, when when unsupported by the unerring guide of experience; and I do not conceive that in 999 cases out of 1000, any of the dreadful consequences would occur, which have so frequently been the concomitants of this disease, when treated agreeably to the dogmas of scholastic instruction.

I am, &c.

*Romsey, Hants, Sept.* 25, 1804. RALPH CUMING.

